# Childhood idiopathic nephrotic syndrome: recent advancements shaping future guidelines

**DOI:** 10.1007/s00467-024-06634-9

**Published:** 2024-12-26

**Authors:** Eugene Yu-hin Chan, Olivia Boyer

**Affiliations:** 1 Department of Paediatrics, Faculty of Medicine, Chinese University of Hong Kong, Shatin, Hong Kong SAR; 2 Paediatric Nephrology Centre, Hong Kong Children’s Hospital, Kowloon, Hong Kong SAR; 3 Néphrologie Pédiatrique, Centre de Référence du Syndrome Néphrotique Idiopathique de L’enfant Et L’adulte, Hôpital Necker - Enfants Malades, APHP, Inserm U1163, Institut Imagine, Université Paris Cité, Paris, France

**Keywords:** Nephrotic syndrome, Pediatric nephrology, Monogenic podocytopathy, Minimal change disease, FSGS, Rituximab

## Abstract

Childhood idiopathic nephrotic syndrome is an important pediatric kidney disease associated with significant morbidities and even mortality. Several guidelines have been developed to standardize the terminology and patient care among the pediatric nephrology community. Since the publication of these guidelines, there have been major breakthroughs in the disease management and the understanding of underlying pathogenesis through multi-omics investigations, including the identification of anti-nephrin autoantibodies, genetic susceptibility loci, and the pathogenic role of B cell subsets. In this educational review, we summarize the recent major advancements in idiopathic nephrotic syndrome and attempt to provide potential therapeutic approaches in both steroid-sensitive and steroid-resistant nephrotic syndrome that may shape future guideline development.

**Graphical abstract:**

**Figure:**
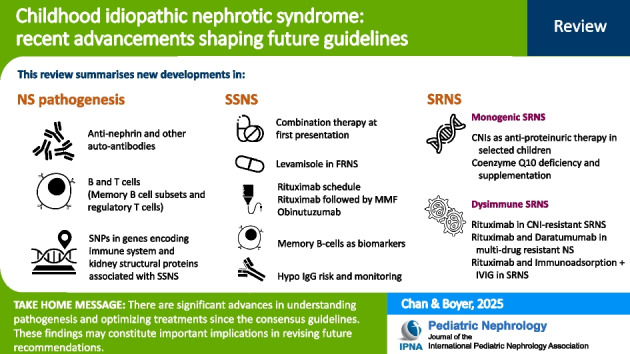
A higher resolution version of the Graphical abstract is available as Supplementary information

**Supplementary Information:**

The online version contains supplementary material available at 10.1007/s00467-024-06634-9.

## Introduction

Idiopathic nephrotic syndrome (INS) is an uncommon childhood condition characterized by edema, heavy proteinuria, hypoalbuminemia, and hyperlipidemia. Although being a rare disease, it is the most common pediatric glomerular disease. A meta-analysis of 27 studies reported a mean incidence of 2.92/100,000 children per year [1, 2]. The reported incidence was stable between 1929 and 2011, where it was higher among Asian children, compared to those originating from North America and Europe (6.1 *versus* 2.4 2.15/100,000 children per year, respectively). The predominant histological diagnosis is minimal change disease in 65% of children, followed by focal segmental glomerulosclerosis (FSGS) (25%) [3]. Among adolescent patients, there is comparable distribution of minimal change disease, FSGS, and other forms of glomerulopathy [3]. In contrast, membranous nephropathy is rare among pediatric population with nephrotic syndrome.

Most cases of INS are steroid-sensitive (SSNS) and can attain complete remission with corticosteroids. Relapses are common and up to 50% of children are either frequently relapsing (FR) and/or steroid-dependent (SD) [4, 5]. Several steroid-sparing agents are utilized, either sequentially or in combination, to sustain disease remission or to reduce the frequency of relapses. A subset of patients however continues to relapse and develop significant medication toxicities, such as Cushing syndrome, short stature, hypertrichosis, and nephrotoxicity. About 10–15% of patients are steroid-resistant (SR), and are at risk of developing kidney failure if they do not respond to further immunosuppressive therapy.

Considerable variations pertaining to the diagnostic criteria and management strategies existed between countries and regions [6]. Consequently, in 2020 and 2022, the International Pediatric Nephrology Association (IPNA) has published the first two clinical practice recommendations in SRNS and SSNS to standardize the terminology and patient care among the pediatric nephrology community [7, 8]. Since the preparation and publication of these guidelines, there have been rapid advancements in the understanding of disease pathogenesis and management over the past few years. The aim of this educational review is to highlight recently available data that may enhance current clinical practice and provide a basis for the development of future guidelines. We will also propose an updated management algorithm based on the new evidence and the existing clinical practice recommendations from IPNA.

## Definitions

The IPNA clinical practice recommendations revise the definition of INS in children in 2020 [8]. The current definition of NS requires both nephrotic-range proteinuria (urinary protein/creatinine ratio (UPCR) 2 mg/mg (≥ 200 mg/mmol)) and a serum albumin of < 30 g/L, instead of 25 g/L, the threshold previously used by several societies. Many acute and chronic pediatric glomerular diseases have similar presentation as NS, including post-infectious glomerulonephritis, IgA nephropathy and IgA vasculitis nephritis, lupus nephritis, and ANCA-associated vasculitis [9, 10]. NS is idiopathic if the aforementioned conditions are ruled out. At present, NS is clinically classified based on steroid sensitivity as SSNS or SRNS, owing to its major management and prognostic implications [11, 12].

## Pathophysiology

### Immune dysregulation and the identification of anti-nephrin autoantibodies

Immune dysregulation is the primary mechanism in the majority of cases with INS, which leads to the production of a circulating glomerular permeability factor. This alters the structure of the podocyte slit-diaphragm, where the main component is nephrin. INS arising from immune dysregulation is mostly steroid-sensitive. However, a subset of children is steroid-resistant and may respond to intensive immunosuppression, or rarely presenting as multi-drug-resistant NS with a high risk of post-transplant recurrence. There is mounting evidence to support the roles of both T and B cell dysregulation in the disease pathogenesis. Single-cell RNA sequencing from blood samples has demonstrated an extrafollicular B cell response during active INS [13]. On the other hand, spatial transcriptomic profiling on kidney biopsies in a subgroup of children with minimal change disease or FSGS shows activation of tumor necrosis factor pathways, which also predicts worse patient outcomes [14].

The identification of anti-nephrin autoantibodies as a potential candidate for circulatory factor in 2022 is the major breakthrough in the understanding of disease pathogenesis [15]. These antibodies were found in both the serum and kidney biopsies of a subgroup of NS patients from the NEPTUNE cohort who had biopsy-proven minimal change disease. At diagnosis, 9/41 children (22%) and 9/21 adults (43%) were positive for anti-nephrin autoantibodies by indirect ELISA, compared to 1/54 disease controls who tested positive for anti-PLA2R autoantibodies. Patients with anti-nephrin autoantibodies also displayed punctate IgG staining on kidney biopsy that co-localized with nephrin. Although the treatment response and relapse-free period were comparable regardless of autoantibody positivity, the autoantibody levels among positive patients were reduced or not detectable during disease remission. The anti-nephrin autoantibody levels were very high before and at the time of transplantation in a patient with immediate post-transplant recurrence. The antibody was no longer detected 1-year post-transplant after complete remission. The role of anti-nephrin autoantibodies in post-transplant recurrence of FSGS was further explored and substantiated in a multi-center Japanese pediatric cohort [16]. In this study, all 11 children with primary FSGS and post-transplant recurrence demonstrated significantly higher levels of circulating anti-nephrin antibodies, compared to subjects with primary FSGS who did not recur, genetic FSGS, and healthy/disease controls. Graft biopsy also showed punctate IgG deposition co-localizing with nephrin. Importantly, plasma exchanges reduced the antibody levels and led to complete disease remission and disappearance of IgG depositions on repeat graft biopsies.

An international, multi-center project further evaluated the role of anti-nephrin autoantibodies in 357 adults with various forms of glomerular diseases and 182 children with INS [17]. Although kidney biopsy was not performed in this pediatric cohort, minimal change disease accounts for roughly 75% of childhood INS [18]. The autoantibodies were detected in 78/98 (80%) children during active NS, and only 1/50 (2%) pediatric controls. Importantly, 35/39 (90%) of children with active NS, who were not receiving immunosuppression at the time of blood sampling, had positive circulating anti-nephrin autoantibodies. In contrast, 46/105 (44%) adults with minimal change disease and 7/74 (9%) subjects with primary FSGS had circulating autoantibodies. The autoantibodies were not found in IgA nephropathy, lupus nephritis, and ANCA-associated vasculitis nephritis, and were only detected in 1/40 (2.5%) adults with secondary FSGS and 1/50 (2%) adults with membranous nephropathy. Serial monitoring of the autoantibodies revealed a close relationship between disease activity and autoantibody levels. Rituximab treatment in two patients (one with relapsing minimal change disease and one with relapsing primary FSGS), who had circulating anti-nephrin autoantibodies, led to clinical remission with a reduction in the antibody levels. These results indicate that anti-nephrin autoantibodies may underlie the immune dysregulation in the majority of pediatric patients and a subset of adults with INS. Furthermore, the autoantibodies appear to play a more important role in the pathogenesis of minimal change disease, compared to primary FSGS, given a higher prevalence of autoantibodies positivity in this specific histological diagnosis. The autoantibody should be added to the growing list of putative circulatory factors such as anti-UHCL1 autoantibodies, hemopexin, CLCF-1, CASK, and suPAR, which may serve as important biomarkers in the near future [2]. Similar to membranous nephropathy, identification of this anti-nephrin autoantibody-associated podocytopathy may enable personalized treatment with anti-CD20 therapy, and guide subsequent medication re-administration.

These results are corroborated by a multi-population GWAS meta-analysis, with 2440 cases of SSNS and 36,023 controls [19]. Single-nucleotide polymorphisms (SNPs) were identified in the *NPHS1* gene, which encodes nephrin, among children presenting with SSNS. Other SNPs were identified in genes related to the immune system (mostly HLA), and those encoding kidney structural proteins which may contribute to the overall genetic architecture of INS [19]. An attractive hypothesis holds that these SNPs could promote immune dysregulation and/or the development of circulating autoantibodies, but this remains speculative and yet to be demonstrated. In contrast, among very rare pedigrees of SSNS, there was no monogenic cause identified even with whole-exome sequencing [19, 20].

### Monogenic podocytopathies

About one-third of children with SRNS have a monogenic cause, which is associated with structural abnormality of the filtration barrier [21]. To date, over a hundred genes have been identified in isolated or syndromic forms of SRNS [22]. There are also considerable ethnic and regional differences pertaining to the causative genetic variants. For instance, in South Africa where the incidence of SRNS is high, up to 55% of pathogenic variants identified were due to homozygous *NPHS2* p.V260E mutation [23]. Autosomal dominant SRNS (*INF2*, *CD2AP*, and *TRPC6*) altogether accounted for 44% of the identified pathogenic variants. In contrast, a multi-center study conducted in China showed that variants in *WT1*, followed by *NPHS1*, *NPHS2*, and *ADCK4*, accounted for 73.6% of children with monogenic SRNS [24]. Importantly, 12.3% of the patients with pathogenic variants carried mutations in *ADCK4*, which functions within the coenzyme Q10 biosynthesis pathway and may warrant targeted therapy. The identification of such genetic causes is of utmost importance for genetic counselling, identification of extra-renal manifestation, limiting unnecessary exposure to immunosuppression, offering specific treatments, selection of intrafamilial kidney donors, and prediction of the risk of post-transplant recurrence.

Taken together, the above exciting findings highly suggest the need to re-characterize patients with INS according to underlying mechanistic pathways, in addition to steroid sensitivity, which may enable truly individualized treatment and ultimately optimize patient outcomes.

## Management of pediatric SSNS

SSNS is defined by attaining complete remission (UPCR < 20 mg/mmol) after 4 weeks or 6 weeks (late responders) of corticosteroids. Prednisolone is subsequently reduced to 40 mg/m^2^/alternate day (max 40 mg/alternate day) for 4 or 6 weeks, then stopped. The IPNA guidelines revised the definition to reflect therapeutic indications [25]. FRNS is now defined by ≥ 2 relapses during the first 6 months or ≥ 3 relapses (previously ≥ 4) in any 12-month period. The rationale of this change is to reduce steroid toxicity secondary to treatment of repeated relapses and to better align with currently accepted indications for steroid-sparing agents [25]. For SDNS, the same definition of ≥ 2 consecutive relapses while on corticosteroids or within 14 days following its discontinuation is used. Since the risk of kidney failure is minimal in pediatric SSNS, the aim of management is to sustain long-term disease control and limit excessive medication side effects.

### Initial therapy at disease presentation

Over the past decade, it has become clear that higher cumulative dose or longer duration of initial steroid treatment does not alter the disease behavior [26, 27]. Recent research directions have since then changed towards limiting treatment-, specifically corticosteroid-, associated toxicities. One important potential strategy is to adopt combination therapy after steroid-induced remission at initial presentation, which is being examined by several trials. Combination therapy was first evaluated by adding an 8-week course of cyclosporine A in the intervention arm, after achieving steroid-induced remission. However, the benefit of this strategy was lost after the medication was discontinued. Preliminary data from a French multi-center randomized placebo-controlled trial, Nephrovir-3 (NCT02818738), showed that add-on levamisole for 6 months, following complete remission, improved relapse-free survival at 12 months by 30% [28]. Combination therapy with mycophenolate mofetil (MMF) to reduce steroid exposure is being investigated by the German INTENT trial, and results are expected to be available in the near future (DRKS0006547) [29]. Another prospective, open-label, single-arm trial evaluated the efficacy and safety of add-on rituximab (single infusion of 375 mg/m^2^) within 1 week after attaining steroid-induced disease remission [30]. Compared to historical controls, the 12-month relapse-free survival was higher (74% vs. 30%) and the incidence of FR/SDNS was also lower at 12 months. Nonetheless, this treatment strategy may expose children to excessive treatment, as 25% will experience a single episode of NS and only 50% patient would develop FR/SDNS, who may be adequately controlled with milder immunosuppression.

### Treatment of NS relapses

Treatment of relapses involves full dose prednisolone or prednisone, given at 60 mg/m^2^/day (max 60 mg/day), until 3 days after complete remission, followed by 40 mg/m^2^/alternate day (max 40 mg/alternate day) for 4 weeks. In 2021, the PROPINE randomized controlled non-inferiority trial demonstrated that progressive steroid tapering (same cumulative prednisolone or prednisone dose but tapered over 40 days (short arm) versus 80 days (long arm)) was not beneficial [31]. Another trial comparing 2 to 4 weeks course of steroids following complete relapse in 117 children also did not show any significant difference in time-to-relapse and relapse rates between these two regimens [32]. However, non-inferiority could not be demonstrated as the study was underpowered. In conclusion, recent data arising from clinical trials do not support the use of prolonged or higher-dose steroid regimens for relapse treatment.

### Steroid-sparing agents

In the current IPNA guidelines, first-line steroid-sparing agents for FR/SDNS are listed according to alphabetical order, including levamisole, MMF, calcineurin inhibitors (CNIs) and cyclophosphamide. The choice of which immunosuppressive agent to be used first has not been agreed. The second-line agents include B cell–depleting, anti-CD20 therapy such as rituximab. The role of therapeutic drug monitoring has also been implicated in medications such as MMF and CNIs, and is extensively reviewed elsewhere [33].

A recent open-label, non-inferiority, randomized controlled trial investigated the efficacy and safety of 12 months of alternate-day prednisolone, given daily during infection, versus levamisole in 160 children with FRNS and/or SDNS [34]. More patients treated in the prednisolone arm developed frequent relapses (40% vs. 22.5%; risk difference 17.5%; 95%CI 3.4–31.6%), and had higher cumulative steroid exposure. Of note, both groups demonstrated similar effects regarding sustained remission, relapse rate, and safety. Details of ANCA-positivity were, however, not adequately evaluated. Although these two strategies both appear to be efficient and safe, levamisole may be favored in children at risk of steroid-toxicity. In view of the established efficacy in preventing relapses in FRNS, low cost and high safety profiles, the role of levamisole in FRNS deserves to be redefined in future guidelines. Early use of levamisole may be considered before prescribing more potent immunosuppression such as MMF and CNIs [35]. Since the drug is not available in some parts of world, there is an advocacy for global access [35].

### Rituximab

Rituximab, a chimeric anti-CD20 monoclonal antibody, has become an important treatment in INS and also many other pediatric glomerular diseases [9, 10, 18, 36–39]. Multiple clinical trials have demonstrated the effectiveness of rituximab in preventing relapses, enabling discontinuation of immunosuppression and improved growth among children with INS [36, 40]. These findings indicate the key role of B cells in the disease pathogenesis. In particular, repopulation of memory B cells, as opposed to total B cells, has emerged as a very useful biomarker [41, 42]. While its reconstitution is more closely associated with disease relapse [41], a higher circulating level of memory B cells prior to rituximab administration also predicts subsequent relapse [42]. Consequently, it would be reasonable to monitor memory B cells, in addition to total B cells, before and after anti-CD20 therapy. In contrast, the presence of anti-rituximab antibodies does not impact relapse rates and probabilities and is not considered a clinically useful biomarker [43].

Patient factor is an important determinant for treatment efficacy of rituximab therapy. Subjects with complicated clinical course, as denoted by history of steroid resistance and higher number of previous immunosuppression use, are more susceptible to relapse [36]. In a recent retrospective study by Colucci et al*.*, older patients > 9.8 years at the time of rituximab administration were less likely to develop a relapse, compared to younger children (HR 0.44, 95% CI 0.26–0.74). The observation that these young patients had a faster and higher recovery of B cell level and its subsets, including memory B cells, may explain the phenomenon. Another explanation is that the natural course of nephrotic syndrome generally improves during and after puberty. Furthermore, the response to rituximab also depends on the dosing regimen and use of concurrent immunosuppression [18]. Previous data had shown that high-dose regimen (375 mg/m^2^ for 4 infusions), originally adopted from the treatment of lymphoma, did not lead to a superior relapse-free survival [18]. While the current IPNA guidelines do not have a specific recommendation on dosing (ranged from 1 to 4 infusions at 375 mg/m^2^), a number of country-specific guidelines/recommendations have proposed to lower the prescription to 1–2 infusions of 375 mg/m^2^ *per treatment course* [44–46].

Up to 80% of children would eventually relapse following rituximab therapy, and require additional course to optimize disease control. Nonetheless, the long-term efficacy and safety of this strategy remains unclear. In an international study, 346 children receiving 2–7 courses of rituximab were analyzed [47]. The median follow-up was 5.9 years. There was a progressive improvement in relapse-free period and relapse risk with repeated courses of rituximab (from 10.0–12.0 to 16.0 months; *P* < 0.001). The side effect profiles also remained similar despite increasing number of courses and cumulative rituximab dose. The main adverse events were hypogammaglobulinemia (50.9%), infection (4.5%), and neutropenia (3.7%), especially among younger children at first rituximab (8.0 versus 10.0 years; *P* = 0.01) [40, 47, 48]. Indeed, hypogammaglobulinemia is common and can occur in 14–58% of cases of INS following rituximab [36, 48, 49]. In a large survey conducted in Europe comprising 1328 children from 84 centers, up to 61% of patients developed hypogammaglobulinemia after rituximab [50]. However, the true risk of developing infection remains unclear, as only 1% of patients developed concomitant infection in the presence of hypogammaglobulinemia after rituximab [47]. Therefore, it is imperative to monitor immunoglobulin levels before and after rituximab, and consider intravenous immunoglobulin for selected indications such as concomitant or repeated infections [36, 51].

A multi-center, randomized, double-blind, placebo-controlled trial evaluated the efficacy of MMF as maintenance therapy after rituximab. Thirty-nine children received MMF and placebo, respectively. There was a clinically but not statistically significant longer time to treatment failure, which was defined as FRNS, SDNS, steroid-resistance, or use of immunosuppressive agents after rituximab (median, 784.0 vs. 472.5 days, *p* = 0.07) [52]. The risk of treatment failure was reduced by 80% (HR, 0.20; 95% CI 0.08–0.50) during MMF maintenance. However, treatment failure was observed in most patients following MMF discontinuation. The frequency and severity of adverse events were comparable between the two arms, where neutropenic episodes were observed in 7.7 and 5.1% of patients. Hypogammaglobulinemia was not reported. Thus, maintenance MMF is a plausible option to sustain disease remission after rituximab and may reduce or delay the need for rituximab re-administration.

Clearly, rituximab is efficacious compared to other oral immunosuppression, and its use may alleviate the concern of medication non-adherence, which is closely associated with disease relapse [53]. However, whether rituximab should be used as first-line steroid-sparing agent remains controversial, especially among young children. One important consideration is the possible sequelae of long-term immunological impairment and reduced vaccine competency [54–57], as well as the currently unknown impact of repeated rituximab administration during childhood when the development and maturation of immunity normally takes place [36].

### Novel therapeutic approaches

A small subset of FR/SDNS patients who are refractory to rituximab and other immunosuppression constitutes the major therapeutic challenge and dilemma for pediatric nephrologists. Rituximab does not efficiently deplete memory B cells residing within tissue and secondary lymphoid organs, such as spleen and lymph node [58, 59], which may continue to produce disease-causing circulating factors. Obinutuzumab, a type II anti-CD20 monoclonal antibody, shows strong homotypic adhesion and potent induction of direct cell apoptosis through non-caspase-dependent pathways. Moreover, obinutuzumab leads to profound B cell depletion and can eliminate B cell and its subsets in both blood and tissue [60]. The superior efficacy of obinutuzumab, in comparison to rituximab, has been demonstrated in adults with membranous nephropathy [61]. Its use has also been shown to be effective as an add-on therapy in lupus nephritis [62]. Dossier et al. recently reported 41 children with rituximab-refractory FR/SDNS treated with a single infusion of obinutuzumab [63]. B cell depletion lasted for a median of 8.3 months, which was longer than that achieved during previous rituximab treatments. Of the subjects, 92% and 68% were in sustained remission at 12 and 24 months. Safety profiles appeared acceptable, with a decrease in IgM but not IgG levels over time. Obinutuzumab has also been reported to sustain long-term remission in a patient who experienced relapse during total circulating B cell depletion [64], which is a significant therapeutic dilemma in rituximab-refractory FR/SDNS.

Belimumab is a monoclonal antibody that inhibits B cell activating factor (BAFF) and is a promising therapeutic option in childhood-onset lupus nephritis [65]. Although mounting evidence supports the role of B cells as the driving force for INS, an open-label, single-arm pilot pediatric study failed to demonstrate a clear benefit of belimumab [66], and the study was terminated early due to the inconvenience of monthly intravenous infusion. Another plausible explanation for rituximab resistance is the contribution of short-lived and long-lived plasma cells to underlying pathogenesis [67]. Unlike other B cell subsets, these cells do not express CD20 marker thus cannot be targeted by anti-CD20 therapy [68]. In this context, add-on daratumumab, an anti-CD38 antibody, in combination with rituximab as a global anti-B cell therapy was recently reported to be an effective approach to overcome rituximab resistance in adolescents and young adults aged between 15 and 24 years [69].

Mesenchymal stromal cells (MSCs) appear to have promising immunomodulatory properties, and have been lately evaluated in children with INS. In a phase I open-label trial, autologous bone marrow–derived MSCs (BM-MSCs) were infused in 10 children with SDNS [70]. Although all children relapsed over a 12-month trial period, there were significantly extended relapse-free survival and reduced relapse number, in comparison to the 12-month period before BM-MCSs infusion. Adverse events appeared to be mild.

## Management of pediatric SRNS

### Dysimmune forms

SRNS is defined by the absence of complete remission after 4–6 weeks of full-dose corticosteroids. CNI for at least 6 months is the treatment of choice in this clinical scenario, coupled with progressive steroid tapering. CNI resistance is defined by the absence of at least partial remission (UPCR > 20 and < 200 mg/mmol, and serum albumin ≥ 30 g/L) after 6 months, where other alternative therapies such as rituximab should be considered. Multi-drug-resistant SRNS is defined by the lack of complete remission following treatment with two different immunosuppressants for 12 months. Children with multi-drug-resistant NS are at high risk of developing kidney failure [71], which is associated with mortality and significant morbidities [71–77]. Thus, it is justified to offer intensive immunosuppression to achieve at least partial disease remission. Renin–angiotensin–aldosterone blockade is also an important adjunct to slow the kidney progression [78, 79].

The efficacy of rituximab as a rescue therapy remains controversial in SRNS [36]. We recently conducted a retrospective cohort study at 28 pediatric nephrology centers from 19 countries [80]. Of the 246 children included, 146 patients were CNI-resistant (≥ 6 months). The remission rates at 6 and 12 months after rituximab were 36% and 35%, respectively. The rest of the cohort (*n* = 100) received rituximab before 6 months’ use of CNIs, due to various reasons such as prolonged hospitalization. These patients had higher rates of remission (6 months, 52%; and 12 months, 55%), likely reflecting the combination effect of rituximab and CNIs. Achieving remission even in a subset of patients is important, as non-remission disease status significantly increases the risk of developing kidney failure. Findings from this study may suggest the potential contribution of circulatory factors, such as anti-nephrin autoantibodies, to the development of SRNS.

Combined rituximab and daratumumab, as described in the aforementioned report [69], was reported to reduce proteinuria in 4 children and 3 adult patients with multi-drug-resistant NS. Among these patients, 4 and 2 patients achieved complete and partial remission following this combination therapy. Five patients developed relapse and were successfully treated with an additional course of therapy. Another potential add-on therapy to anti-CD20 monoclonal antibodies is immunoadsorption and intravenous immunoglobulins. In a case series, 14 patients were treated with 10 sessions of immunoadsorption over 2 weeks, with intravenous immunoglobulin and a single infusion of rituximab given at 375 mg/m^2^ [81]. Remission of proteinuria was observed in 11 patients.

Lastly, a prospective, open-label, single-arm phase 1–2 pilot study evaluated 11 pediatric patients with multi-drug refractory INS who received allogenic cord blood-derived MSCs intravenously [82]. Of the 9 patients available for efficacy assessment, 3 patients attained either complete or partial remission. There was no reported adverse event or toxicity. Future trials are required to identify and establish the roles of new therapeutic options.

## Monogenic SRNS

Children with monogenic SRNS are less likely to respond to immunosuppressive therapy than those with a negative genetic test [11, 79]. A multi-center cohort showed that only 1/26 (4%) of patients with monogenic SRNS attained complete remission with immunosuppression, while remission was achieved in one fourth of children with negative genetic tests (*n* = 37/149) (*p* = 0.02).

Therefore, CNI is not routinely recommended for children with monogenic SRNS [8]. This treatment was recently re-visited in an international multi-ethnic cohort of 141 children with monogenic SRNS [83]. Favorable outcomes were reported, in which 6 months of treatment with CNIs was associated with complete remission in 6.3% and partial remission in 21.3%, even though there may be a confounding effect from renin–angiotensin–aldosterone blockade. The reduction in proteinuria has been attributed to the drug’s non-immunologic effects including afferent arteriole vasoconstriction and, potentially, stabilization of the podocyte cytoskeleton. Higher baseline albumin predicted favorable response and a positive response to CNIs significantly reduced the risk of reaching kidney failure. Conversely, no child with congenital nephrotic syndrome responded to treatment. Therefore, a short trial of CNI may be considered in selected patients.

Identification of underlying genetic causes may enable personalized treatment. Coenzyme-Q10 deficiency, among patients with biallelic variants in *COQ2*, *COQ6*, *PDSS2*, and *COQ8B,* is the first illustrative example of this. An international study demonstrated that coenzyme-Q10 supplementation reduced proteinuria by 88% at 12 months in 41 children, compared to an untreated matched-control group [84]. Some of the treated patients might occasionally achieve complete remission. Coenzyme-Q10 supplementation also led to significantly better 5-year kidney failure-free survival (62% vs. 19%).

### APOL1-mediated SRNS

Inaxaplin, an orally active apolipoprotein 1 function inhibitor for proteinuric diseases, has been an important advancement for personalized medicine among patients with *APOL1* variants. At-risk *APOL1* variants (G1/G2) are enriched in West-African and West-Indian populations due to trypanosomiasis resistance. The presence of these variants in a bi-allelic state demonstrates a faster rate of progression to kidney failure, although sensitivity to steroids and other forms of immunosuppression was comparable to other subjects. A phase 3 clinical trial examined the effect of inaxaplin in 16 adults with a mean proteinuria of 2 mg/mg at baseline [85]. Proteinuria decreased by 50% after 13 weeks of treatment. Adverse events were mostly mild. It could well become the second personalized treatment in genetic SRNS following ubiquinone in coenzyme-Q10 deficiency.

### Transplantation

Post-transplant NS recurrence is a severe complication which is significantly associated with early graft loss. A meta-analysis comprising 8 studies and a total of 581 children with SRNS concluded that the overall post-transplant recurrence rate was 39% [86]. None of the patients with monogenic SRNS had disease recurrence. In contrast, up to 61% of children without identified genetic variants developed a relapse post-transplant. Importantly, late steroid resistance, compared to initial steroid resistance, doubled the risk of developing NS recurrence post-transplant (1.91; 95% CI: 1.48–2.46), which concurs with previous reports [87].

## Outcomes of NS

Patient outcomes have changed dramatically over the past two decades with advancements in immunosuppression. Infectious complications due to peritonitis and bacteremia are rarely observed in the modern era [88]. In a multi-center Japanese study involving 187 children with INS, only 5 subjects developed these complications. Instead, treatment-associated infection such as pneumonia was prevalent (95.5 episodes per 1000-patient-year), especially after rituximab therapy (318 episodes per 1000-patient-year) [88]. Fortunately, these children are not at significant risk of developing severe COVID-19 infection, with or without immunosuppression use [89].

Malignant potential remains to be a concern following the protracted use of immunosuppression. Over a period of 20 years, the incidence of lymphoproliferation was reported to be 5 per 1000-patient-year in a multi-center French study [90]. These included Hodgkin lymphoma and lymphoproliferative disease, and were associated with viral infections such as EBV or HHV-8 [90]. Although there were isolated reports of neoplasm after rituximab, it was found to be extremely rare (0.1%) in the aforementioned international cohort where repeated courses of rituximab were given for INS [47]. Nonetheless, one important caveat in interpreting safety profile of rituximab is that most data available come from children older than 6 years of age with follow-up duration less than 5 years [36].

Obesity and short stature are not uncommon in INS. In a large prospective study which analyzed a total of 531 children with INS, the cumulative incidence of new-onset obesity and short stature were 17.7% and 3.3%, respectively [91]. Nonetheless, these rates are believed to be lower in the modern era with effective immunosuppression that prevents multiple relapses and steroid exposure. Finally, it should be remembered that while most children and adolescents with NS have normal kidney function (i.e., CKD stage 1), the disease can lead to poor health-related quality of life and emotional and behavioral difficulties as well as chronic fatigue, with long-term negative psycho-social impact into adulthood [92, 93].

These results reiterate the importance of judicious and rational use of immunosuppression, balancing disease control and treatment side effects, to optimize long-term patient outcomes and restore normal childhood in children suffering from INS.

## Conclusion and the way forward

**Fig. 1:**
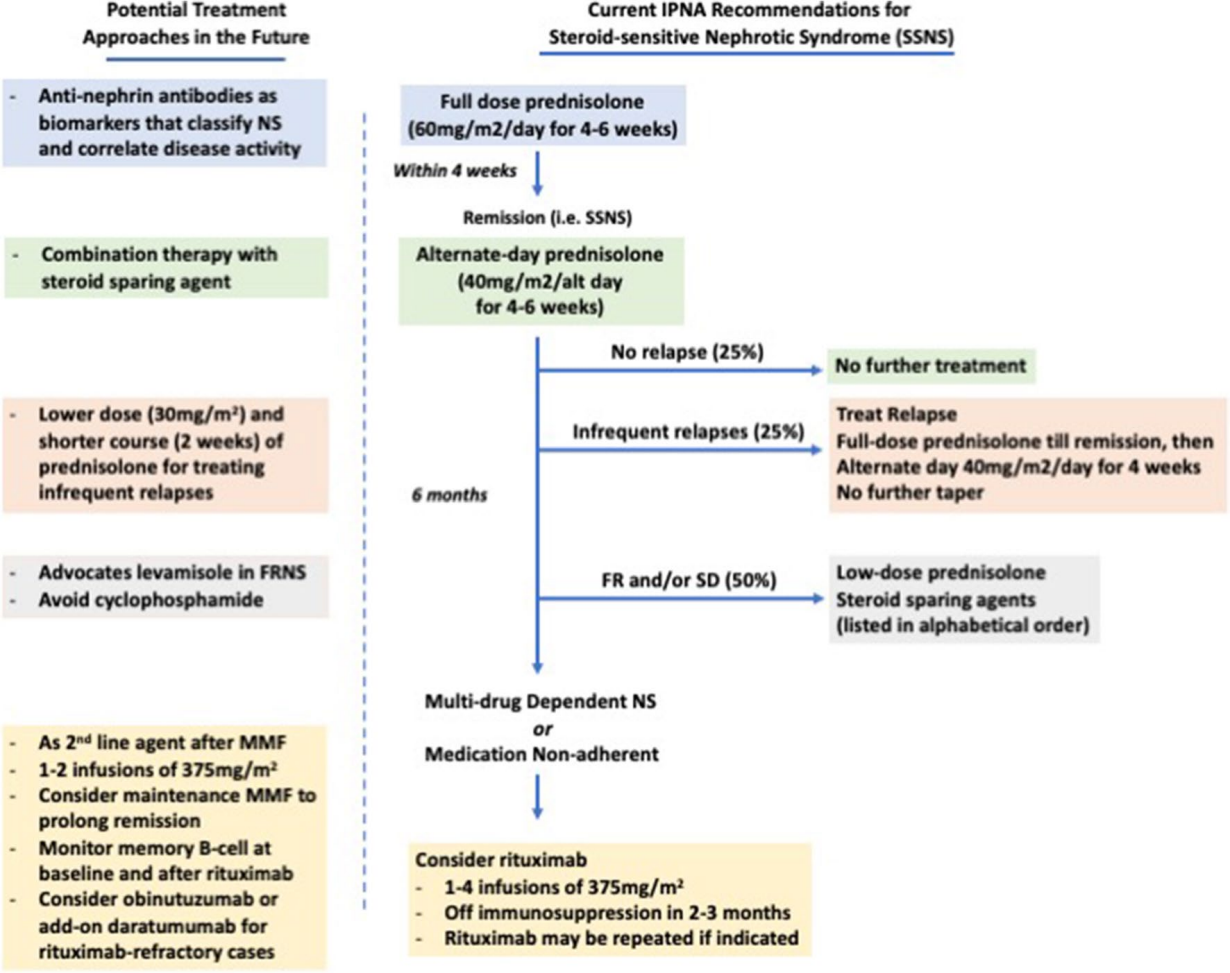
Current IPNA recommendations and potential therapeutic treatment approaches for steroid-sensitive nephrotic syndrome (SSNS). FR, frequently relapsing; MMF, mycophenolate mofetil; SD, steroid-dependent

**Fig. 2:**
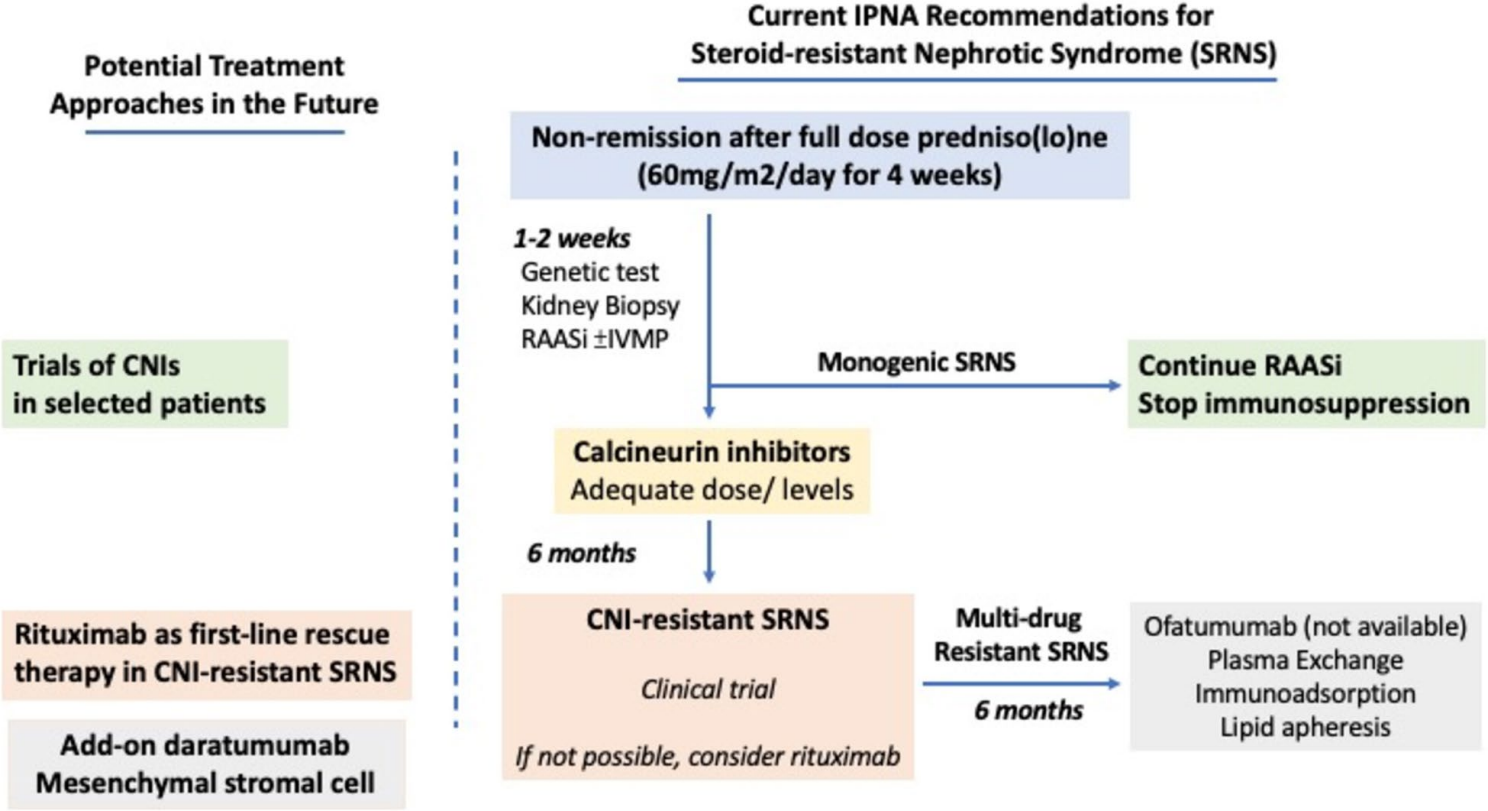
Current IPNA recommendations and potential therapeutic treatment approaches for steroid-resistant nephrotic syndrome (SRNS). CNIs, calcineurin inhibitors; IVMP, intravenous pulse methylprednisolone; RAASi, renin–angiotensin–aldosterone-system inhibitors

Significant advances in understanding disease pathogenesis and optimizing treatment outcomes have been achieved since the publication of consensus guidelines. These findings may constitute important implications in revising future guidance, and are summarized in Figs. 1 and 2. In particular, the current classification of INS is clinical, and based on treatment response to immunosuppression and the presence of genetic variants. Patterns of kidney injury on kidney histology also provide poor correlation with underlying pathophysiology. In the future, pragmatic classification addressing specific mechanistic pathways should be considered to enable personalized and precision medicine This effort will hopefully translate into improved long-term patient outcomes.

## Supplementary information

Below is the link to the electronic supplementary material.

Graphical abstract (PPTX 130 KB)
