# Supplementary material for: Childhood idiopathic nephrotic syndrome: recent advancements shaping future guidelines
Source: Pediatr Nephrol. 2024 Dec 26;40(8):2431–42. doi: 10.1007/s00467-024-06634-9 (PMC12187818; doi:10.1007/s00467-024-06634-9)
Supplement: Supplementary file 1 — Graphical abstract (PPTX 130 KB) [file 467_2024_6634_MOESM1_ESM.pptx]

## Slide 1
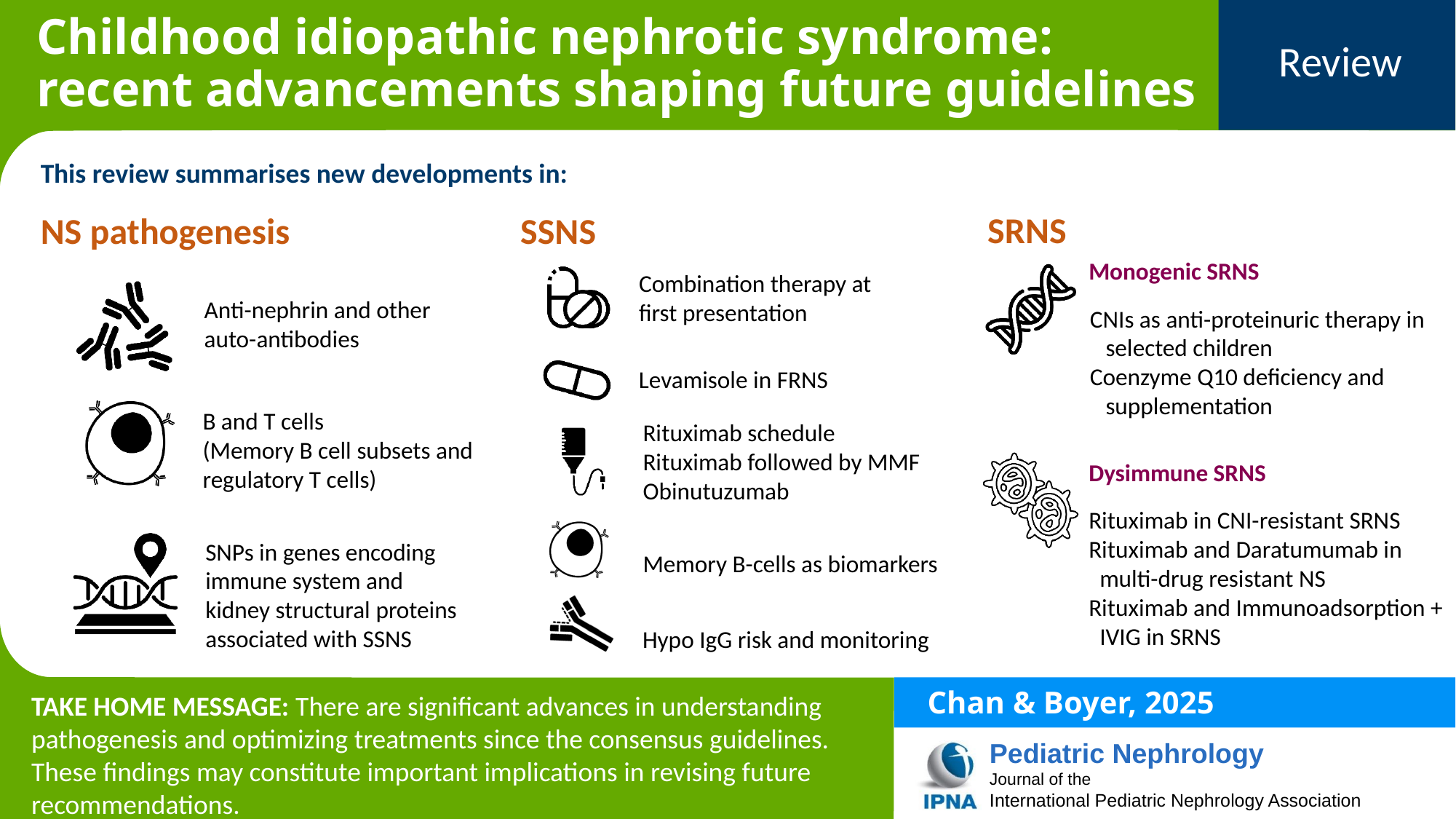

Childhood idiopathic nephrotic syndrome:
recent advancements shaping future guidelines
This review summarises new developments in:
SRNS
NS pathogenesis
SSNS
Monogenic SRNS
CNIs as anti-proteinuric therapy in selected children
Coenzyme Q10 deficiency and supplementation
Combination therapy at
first presentation
Anti-nephrin and other
auto-antibodies
Levamisole in FRNS
B and T cells
(Memory B cell subsets and regulatory T cells)
Rituximab schedule
Rituximab followed by MMF
Obinutuzumab
Memory B-cells as biomarkers
Dysimmune SRNS
Rituximab in CNI-resistant SRNS
Rituximab and Daratumumab in multi-drug resistant NS
Rituximab and Immunoadsorption + IVIG in SRNS
SNPs in genes encoding
immune system and
kidney structural proteins associated with SSNS
Hypo IgG risk and monitoring
Chan & Boyer, 2025
TAKE HOME MESSAGE: There are significant advances in understanding pathogenesis and optimizing treatments since the consensus guidelines. These findings may constitute important implications in revising future recommendations.
